# Pollen-Food Allergy Syndrome in Children: Global Prevalence and Pathogenesis

**DOI:** 10.3390/nu18142246

**Published:** 2026-07-09

**Authors:** Aleksandra Marzec, Dominika Mysiorska, Julia Młyńska, Anna Nowak-Wegrzyn, Urszula Jedynak-Wąsowicz, Elżbieta Jarocka-Cyrta

**Affiliations:** 1 Department of Clinical Pediatrics, Medical School, University of Warmia and Mazury in Olsztyn, 10-561 Olsztyn, Poland; dominikamysiorska@gmail.com (D.M.); juliamlynska00@gmail.com (J.M.); anna.nowak-wegrzyn@nyulangone.org (A.N.-W.); ejarocka@op.pl (E.J.-C.); 2 Department of Pediatrics, Gastroenterology and Nutrition, Provincial Specialist Children’s Hospital in Olsztyn, 10-561 Olsztyn, Poland; 3 Doctoral School, Nicolaus Copernicus Superior School, 31-150 Warsaw, Poland; 4 Division of Allergy and Immunology, Department of Pediatrics, Hassenfeld Children’s Hospital, NYU Langone Health, New York University R. Grossman School of Medicine, New York, NY 11042, USA; 5 Clinic of Pediatrics, Jagiellonian University Medical College, 30-663 Krakow, Poland; ula.jedynak-wasowicz@uj.edu.pl; 6 Department of Pulmonology, Allergy and Dermatology, Children’s University Hospital, 30-663 Krakow, Poland

**Keywords:** pollen–food allergy syndrome, oral allergy syndrome, children, pediatric allergology, epidemiology, cross-reactivity

## Abstract

Pollen–food allergy syndrome is an IgE-mediated allergic condition resulting from cross-reactivity between pollen allergens and homologous proteins present in plant-derived foods. PFAS typically presents with rapid-onset itching, burning, swelling, and erythema of the oral cavity and pharynx, with symptoms usually limited to the oropharyngeal region, although systemic reactions, including respiratory or gastrointestinal symptoms and, rarely, anaphylaxis, may occur. PFAS is strongly associated with the atopic phenotype and frequently coexists with allergic rhinitis, atopic dermatitis, and asthma. Available studies show marked variability in prevalence, from a few percent in general pediatric populations to 50–80% among atopic children, especially those with allergic rhinitis, with regional differences likely reflecting pollen sensitization patterns and dietary habits. The aim of this review was to summarize current knowledge regarding the epidemiology, clinical characteristics, molecular determinants, and food-related aspects of PFAS in children across different regions of the world. PFAS is a common but still insufficiently studied condition in children. Standardized diagnostic criteria and large population-based studies incorporating molecular diagnostic approaches are needed to better define the true burden, risk factors, and clinical spectrum of PFAS in childhood.

## 1. Introduction

Pollen–food allergy syndrome (PFAS) is an IgE-mediated allergic condition caused by cross-reactivity between pollen allergens and homologous food proteins. It is characterized by immediate, transient symptoms primarily involving the oral mucosa that occur upon ingestion of certain fruits, vegetables, and nuts.

Common clinical manifestations of PFAS include itching, burning sensations, swelling, and erythema affecting the mouth, lips, and throat shortly after ingestion of raw fruits, vegetables, nuts, or spices. In most cases, symptoms are confined to the oral cavity, as the acidic environment of the stomach efficiently inactivates the responsible allergens and terminates the reaction. However, in rare situations, symptoms may develop into systemic reactions, including urticaria, angioedema, airway swelling, cough, dyspnea, vomiting, abdominal pain, and, in severe cases, anaphylaxis.

Regional differences in PFAS-triggering foods reflect variations in local vegetation and dietary habits. In Northern Europe, PFAS predominantly affects individuals sensitized to birch pollen, whereas in East Asia, sensitization patterns are primarily driven by regional tree pollen such as Japanese cedar, cypress, pine, and alder. Across regions, commonly implicated foods include apples, peaches, cherries, celery, carrots, tomatoes, walnuts, hazelnuts, almonds, and soy products [1,2].

The diagnosis of pollen–food allergy syndrome (PFAS) is primarily based on a detailed clinical history supported by allergy testing. Component-resolved diagnostics (CRD) plays a pivotal role in differentiating primary food sensitization from pollen-related cross-reactivity, as well as in assessing the underlying immunological mechanisms and the risk of systemic reactions, particularly in patients sensitized to stable allergenic proteins that are resistant to heat and enzymatic digestion [2]. Although oral food challenges remain the diagnostic gold standard, they are often unnecessary in patients with a consistent clinical history and concordant molecular diagnostic findings.

Management of PFAS mainly involves avoiding raw trigger foods, as symptoms are usually mild and limited to the oral cavity. Most allergens are heat-labile, so cooked or processed foods are typically tolerated. Dietary management should be individualized to avoid unnecessary restrictions, and antihistamines are effective for mild symptoms, while epinephrine is indicated in patients at risk of anaphylaxis [3].

PFAS remains insufficiently characterized in the pediatric population despite its growing clinical relevance. This knowledge gap may be attributed to underrecognition of mild and transient symptoms, limited symptom reporting by children and caregivers, and methodological limitations of existing studies, many of which rely predominantly on questionnaire-based data. Moreover, the differentiation between primary food allergy and pollen-related cross-reactivity often requires component-resolved diagnostics, which is not uniformly available in routine clinical practice.

The aim of this review is to summarize current knowledge on the epidemiology, clinical characteristics, and mechanisms of pollen–food allergy syndrome in pediatric populations. PFAS phenotypes are shaped by regional pollen exposure, dietary patterns, and molecular sensitization profiles. A comprehensive synthesis may improve clinical recognition, support risk stratification, reduce unnecessary dietary restrictions, and guide future research priorities in pediatric allergy.

## 2. Pathogenesis

PFAS results from cross-reactivity between pollen allergens and structurally homologous food proteins sharing similar epitopes, whereby pollen-specific IgE recognizes homologous proteins in plant-derived foods and triggers allergic reactions. Such cross-reactivity generally requires a high degree of structural homology, often exceeding 70% sequence similarity. It typically develops after primary sensitization to pollen via the respiratory tract; in most cases, symptoms of pollen allergy, particularly pollen-induced allergic rhinitis, precede the onset of food-related reactions. However, some children may have no overt rhinitis despite evidence of pollen sensitization on skin prick testing or serum-specific IgE assays. Subsequent ingestion of raw plant foods may then trigger reactions to cross-reactive proteins, even without prior exposure to the culprit food. Not all pollen-sensitized children develop PFAS, and clinical reactivity may involve one or multiple foods [4].

In the development of PFAS, class II food allergens play a central role and differ from class I allergens in their route of sensitization, biochemical stability, and clinical relevance. Class I food allergens induce primary sensitization via the gastrointestinal tract, are resistant to heat and digestion, and often cause systemic reactions. In contrast, class II allergens act through cross-reactivity in pollen-sensitized individuals, are labile to heat and digestion, and typically cause mild, localized oral symptoms, often disappearing after cooking [5]. The distinction is presented in Table 1 [6].

The molecular basis of PFAS is largely attributed to several families of widely distributed plant panallergens. Panallergens are structurally conserved proteins that fulfill important biological functions in plants while playing a key role in the development of cross-reactive IgE-mediated allergies and determining the clinical severity of allergic reactions. The most important group in Central and Northern Europe comprises PR-10 proteins, homologues of the major birch pollen allergen Bet v 1, including Mal d 1 in apple, Pru p 1 in peach, Api g 1 in celery, Ara h 8 in peanut and Cor a 1 in hazelnut. Although PR-10 proteins are generally labile, some members, such as those found in celery and soybean (Gly m 4), may occasionally induce systemic reactions [7,8]. Profilins (Bet v 2) represent another panallergen family, present in nearly all eukaryotic cells and responsible for broad cross-reactivity between pollens and numerous fruits such as melon, banana, and citrus; however, their marked thermolability usually limits clinical symptoms to the oral cavity. Lipid transfer proteins (LTPs, PR-14) constitute an exception, as they may function as both class I and class II allergens. Due to their high structural stability, they are frequently associated with severe systemic reactions and may cause either primary food allergy (e.g., Pru p 3 in peach) or pollen-related cross-reactivity (e.g., with mugwort Art v 3). Recently, gibberellin-regulated proteins (GRPs), such as Pru p 7 in peach, have been identified as stable allergens capable of cross-reacting with cypress or cedar pollen and inducing severe clinical manifestations. Finally, cross-reactive carbohydrate determinants (CCDs) may cause extensive serological cross-reactivity, although they are generally of limited clinical relevance [3].

### PFAS vs. OAS

In medical terminology, both Oral Allergy Syndrome (OAS) and Pollen–Food Allergy Syndrome (PFAS) are used, often interchangeably, although they describe related but distinct concepts. OAS describes a phenotype based on the constellation of symptoms limited to the oropharynx, whereas PFAS defines an endotype, based on the mechanism—a specific form of IgE-mediated food allergy in which primary sensitization occurs through pollen exposure. In PFAS, OAS symptoms arise due to cross-reactivity between pollen allergens and homologous plant food proteins, most commonly in patients with allergic rhinitis. According to current concepts, the term OAS is considered insufficiently precise and potentially misleading, as it may imply a uniformly mild clinical course and thus contribute to underestimation of the risk of systemic reactions in some patients. Consequently, for food reactions associated with pollen sensitization- particularly those involving fruits and vegetables—the more specific term PFAS has been recommended [9].

In this review, some studies describe PFAS, while others address OAS; several also distinguish between the two entities and report on both conditions. The distinction is presented in Table 2.

## 3. Methods

We conducted a scoping review of the literature published between 1 January 2000 and 28 February 2026, identified through searches of PubMed/MEDLINE, Scopus, Embase, and Google Scholar. The search strategy combined controlled vocabulary terms and free-text keywords related to pollen–food allergy syndrome and pediatric populations. The following keywords were used alone and in combination: “pollen-food allergy syndrome,” “PFAS,” “oral allergy syndrome,” “OAS,” “pollen-related food allergy,” “children,” “pediatric,” “adolescent,” “prevalence,” “epidemiology”.

Eligible articles included original studies, cohort studies, cross-sectional studies, case series, and relevant reviews addressing the epidemiology, clinical presentation, allergen profile, pathogenesis, diagnosis, or management of PFAS/OAS in children and adolescents. Studies focused exclusively on adults were excluded unless they contained pediatric subgroup data relevant to the review objectives. We also excluded conference abstracts without sufficient data, duplicate records, and studies not directly addressing PFAS or OAS in the context of pollen sensitization.

After the removal of duplicates, titles and abstracts were screened for relevance. Full texts of potentially eligible articles were then assessed. Data were extracted narratively with particular attention to study setting, population characteristics, diagnostic definitions, prevalence estimates, reported symptoms, associated pollen and food allergens, atopic comorbidities, and major diagnostic or pathogenetic findings. Because of the substantial heterogeneity in study design, case definitions, and outcome reporting, the results were synthesized descriptively rather than quantitatively.

## 4. Results

We identified 38 studies meeting the inclusion criteria; 16 were excluded and the final analysis included 22 articles.

## 5. Epidemiology

Epidemiological data on PFAS in pediatric populations remain underrepresented in the current literature. Most of the available evidence derives from Asia, while only a limited number of studies have been conducted in Europe, and single investigations are available from Mexico, the United States, and Australia. Furthermore, most of the studies are based on cohorts with allergic diseases, whereas investigations conducted in the general pediatric population are less common. Some studies assessed only the prevalence of OAS symptoms by comparing them with the occurrence of allergic disease symptoms, without using laboratory methods to confirm the diagnosis, which complicates the interpretation of the findings. As the choice of study population is critical for interpretation, the lack of general population–based studies in children limits the accurate assessment of the overall impact of PFAS. Nevertheless, evidence from both general population studies and clinical subgroups indicates that PFAS is more prevalent than previously recognized, highlighting its global significance and the urgent need for dedicated pediatric epidemiological research.

### 5.1. General Population vs. Atopic Patient Cohorts

The prevalence of PFAS demonstrates considerable variability, ranging from approximately 3% [10] to 80% [11], in pediatric and adolescent populations, depending on the study population (general cohorts vs. patients with atopic diseases) and geographic region.

In population-based cohort and survey studies, prevalence estimates typically range from 4.7% [12] to 15.6% [12]. The lowest rate was reported in the Korean COCOA cohort (4.7% among children aged 6–10 years) [12]. In this study, OAS was identified based on a questionnaire-based assessment. Although diagnostic testing was performed, it was not intended to confirm cross-reactive allergy; rather, it aimed to evaluate the presence of allergic conditions, including sensitization to the most common inhalant allergens. The prevalence of OAS was 7.2% in children with AR and increased to 19.1% in those with pollinosis. The highest prevalence was observed in a multicenter study conducted in four Japanese cities (15.6% among children aged 7–15 years) [13]. A total of 524 children (15.6% of all participants) reported OAS-like symptoms, of whom 313 (59.7%) had a diagnosis of seasonal AR. In the most recent cross-sectional study based on questionnaires administered to elementary and junior high school students in Omihachiman, Japan, the overall prevalence of oral symptoms suggestive of OAS was 12.4% [14].

In studies involving atopic populations, particularly children with AR or AD, the prevalence is consistently elevated, typically ranging from 20% to 50%. In London, PFAS was identified in 48% of children with seasonal AR, with rates rising to 78% among adolescents aged 11–15 years [11]. In a Korean questionnaire-based study of patients with pollen sensitization, the prevalence of PFAS in the pediatric subgroup was 42.7% [15]. The study population included individuals diagnosed with allergic rhinitis (AR), allergic conjunctivitis (AC), and/or bronchial asthma (BA), with sensitization to pollen from one or more trees, grasses, and/or weeds. In the same study, the prevalence among adult patients was 40.8%. Significantly lower prevalence of PFAS (6.9%) was observed in a questionnaire-based study conducted among children aged 6–15 years in Saitama, Japan, where PFAS was defined as an allergy to fruits and/or vegetables that developed after the onset of AR symptoms [16]. In a cohort of children with seasonal allergic rhinitis caused by plant pollens and confirmed by skin prick testing in Ankara, Turkey, symptoms of PFAS were identified in only 3.3% of participants, representing the lowest prevalence reported in the atopic population [17].

Overall, PFAS prevalence varies considerably depending on the studied population. Rates are consistently higher among children with pollen sensitization and allergic rhinitis than in general pediatric cohorts. Geographic variability largely reflects regional pollen exposure patterns and local dietary habits.

### 5.2. Clinical Characteristics of PFAS in Children

#### Oropharyngeal Symptoms

Oropharyngeal symptoms dominate the clinical presentation and are reported in the vast majority of pediatric patients with PFAS, with most children experiencing exclusive oropharyngeal manifestations. They result from direct contact between food allergens and mast cells in the oral mucosa and represent a form of contact urticaria with rapid onset, usually occurring immediately or within minutes after ingestion. In numerous studies, the presence of oropharyngeal symptoms constituted a diagnostic criterion for PFAS and served as an inclusion requirement for subsequent analyses. The most common oropharyngeal symptoms are oral itching and tingling [16,18,19,20], whereas the least frequent manifestations within the oral cavity and pharynx include lip swelling and local ear and nasal symptoms. In studies that examined symptom profiles within pediatric PFAS populations, distinguishing local from systemic manifestations, oropharyngeal symptoms were consistently predominant, although not universal, as a subset of patients reported systemic involvement (e.g., cutaneous or respiratory symptoms). In a study conducted in Saitama [16], oral symptoms were reported by 83.9% of children with PFAS, while throat symptoms occurred in 49.4%. Oral pruritus or pain represented the most frequent local manifestation, and nearly 70% of affected children experienced symptoms confined to local sites, including the oral cavity, throat, eyes, nose, or ears. In a cohort of adolescents from Tokyo itching of the mouth and throat was the most reported symptom, affecting over 80% of patients [18]. Consistent findings were observed in a Mexican pediatric cohort where oral tingling or irritation and oral or throat pruritus were the most frequently reported symptoms, while systemic manifestations were rare, occurring in only one patient [20].

PFAS-related oral symptoms may occur year-round despite the seasonal pattern of respiratory allergic manifestations. The study by Westman et al. demonstrated that although the period of peak rhinitis symptoms is relatively short and largely confined to the pollen season—particularly in patients sensitized to a single pollen group such as deciduous trees, grasses, or weeds—oral symptoms triggered by pollen-related food allergens may persist throughout the year [21]. This persistent symptomatology in birch-sensitized children reflects the cross-reactive nature of Bet v 1-related allergens, as homologous proteins present in various plant-derived foods can elicit reactions independently of seasonal pollen exposure. The symptoms are usually most prominent during or immediately after the pollen season [18].

### 5.3. Extra-Oropharyngeal Symptom and Systemic Manifestations

Although PFAS has traditionally been regarded as a condition limited to the oropharyngeal area, evidence indicates that many children develop extra-oral or systemic manifestations, reflecting a broader clinical spectrum. This may occur when the amount of ingested raw food exceeds the digestive capacity of the stomach, allowing intact or only partially degraded allergens to be absorbed and trigger systemic allergic symptoms. Population-based studies, including birth cohorts and large school-based surveys, consistently demonstrate a relatively low prevalence of systemic reactions among patients with PFAS, ranging from 3.8% up to 30.1%, largely reflecting differences in case definitions and recruitment strategies [13,16,22]. Cutaneous symptoms (particularly urticaria and pruritus) and subsequently respiratory symptoms are by far the most reported systemic reactions, regardless of whether general or clinical populations are studied.

The prevalence of systemic reactions is higher, particularly in studies focusing on patients with confirmed pollen sensitization and broad dietary exposure. In a Korean study of patients with pollinosis, generalized symptoms were reported as follows: cutaneous symptoms in 43.0%, respiratory symptoms in 20.0%, gastrointestinal symptoms in 10.7%, neurological symptoms in 4.8%, and cardiovascular symptoms in 3.7% [15]. In a Japanese survey of schoolchildren, 30.1% of PFAS patients reported systemic symptoms beyond the oral cavity [16]. In contrast, the occurrence of systemic reactions is low in specific populations. In Croatia, only one of 120 children with PFAS (<1%) reported systemic complaints, limited to nausea and abdominal pain [19], while in Mexico, a single case (4.2%) involved a rash [20]. These regional differences may reflect genetic background, pollen sensitization profiles, or methodological variations in study design, but collectively they highlight that systemic PFAS manifestations are clinically relevant, even if not universally observed.

### 5.4. Risk of Anaphylaxis

While PFAS is usually mild, severe systemic reactions, including anaphylaxis, can occur. The most common risk factors for severe systemic reactions in PFAS include sensitization to stable allergens, particularly LTPs and GRPs, reactions to processed foods, high allergen dose, especially in liquid form, and the presence of cofactors such as exercise, alcohol, acid-suppressive therapy, NSAIDs, uncontrolled asthma, high pollen exposure, and a history of previous systemic reactions [23]. In the Saitama cohort, the prevalence was 5.8%, and anaphylaxis was defined as the presence of relatively severe symptoms involving more than two organ systems, which may have excluded milder manifestations [16]. As the questionnaire assessed only the presence or absence of symptoms without accounting for their severity, the prevalence of anaphylaxis may have been overestimated, and this limitation should be considered when interpreting the results. In the Korean study [15], 8.9% of PFAS patients experienced anaphylaxis, and similarly, a Turkish study in children with seasonal allergic rhinitis reported anaphylaxis in 9% of cases after exposure to the suspected food allergen [17]; however, in both studies, the authors did not provide a definition of anaphylaxis. The most common triggers of anaphylaxis differed across regions, with Chinese bellflower root and crown daisy reported in Korea, cherry in Japan, and peach in Turkey.

Longitudinal data from the United States demonstrate evolving perceptions of anaphylaxis risk in PFAS. Between 2003 and 2023, the proportion of allergists reporting systemic reactions in their patients increased from 20% to 67%, while the estimated percentage of affected patients decreased from 5% to 1%, suggesting improved recognition of a smaller high-risk subgroup. Awareness that PFAS may present with severe symptoms has also increased, and tree nuts and peanuts are now reported nearly as often as fruits as triggers of systemic reactions, raising clinical concern due to their association with more severe outcomes [9,24].

These findings underscore that PFAS cannot be regarded as universally benign. Consequently, current recommendations emphasize that children with generalized reactions or a history of anaphylaxis should avoid culprit foods strictly and be equipped with an emergency kit, including an adrenaline auto-injector, antihistamines [25,26].

### 5.5. Characterization of Pollen and Food Allergens in PFAS in Children

The development of PFAS in children results from cross-reactivity between pollen allergens and homologous proteins present in fruits, vegetables, and nuts. Epidemiological analyses indicate that the profile of pollen sensitization and associated food allergens varies significantly across regions.

### 5.6. Pollen Allergens

#### Birch Homologous Group (Betulaceae Family)

One of the most extensively studied sources of cross-reactivity in PFAS involves pollen from the Betulaceae family, such as birch pollen and alder pollen.

Birch pollen is one of the most important inhalant allergens responsible for the development of PFAS worldwide. The principal sensitizing component of birch pollen is Bet v 1, a protein belonging to the pathogenesis-related protein 10 (PR-10) family. Bet v 1 exhibits high structural homology with proteins present in numerous fruits, vegetables, and nuts, which explains the frequent occurrence of cross-reactive allergic symptoms following food ingestion in birch pollen–sensitized individuals. Elevated sIgE concentrations against birch or Bet v 1 represent a significant risk factor for the development of PFAS. Patients sensitized to birch pollen most commonly experience allergic reactions after consumption of foods from the Rosaceae family, particularly apple, which is the most frequently reported trigger. Other commonly implicated foods include peach, pear, cherry, plum, strawberry, carrot, celery, potato, hazelnut, peanut, and soy [1,5,9].

Regional variability in birch pollen allergy prevalence reflects differences in local flora, with particularly high rates observed in Northern and Central Europe, moderate prevalence in East Asia, and very low prevalence in regions such as Mexico [20]. A longitudinal study conducted in Paris demonstrated a substantial increase in birch pollen allergy over a 25-year period among children with asthma [27]. In a Swedish study, 31% of children sensitized to birch pollen presented symptoms of PFAS. Longitudinal cohort data further indicate that sensitization to birch pollen often precedes the development of allergic rhinitis and PFAS, and that a substantial proportion of birch-sensitized children subsequently go on to develop food-related allergic symptoms consistent with PFAS [21]. In a study from Zagreb involving children with seasonal allergic rhinitis, sensitization to tree pollen was observed in 79.2% of participants, with birch pollen identified as the allergen most strongly associated with more severe OAS symptoms [19]. Similarly, a study from London reported tree pollen sensitization in 76% of children with seasonal allergic rhinitis; 92% were sensitized to the major birch allergen Bet v 1 [11].

In geographic regions where birch pollen exposure is limited, such as parts of Japan and Korea, alder pollen may serve as the primary sensitizing agent. In Japanese children and adolescents, alder sensitization was linked to a six-fold higher risk of PFAS [28]. Korean studies similarly highlighted elevated sensitization rates to alder among PFAS patients [15]. Regional data from Busan (South Korea), Fukuoka, and Tochigi (Japan) consistently reinforced the importance of alder as a key trigger of PFAS. Additional Betulaceae species, such as beech and hazel, also showed higher sensitization rates among PFAS patients in Korea [29] PR-10 proteins are generally heat-labile; therefore, thermal processing such as cooking or baking often reduces or eliminates their allergenicity [30]. However, patients with high levels of birch-specific IgE and concomitant atopic dermatitis may experience more severe manifestations, including exacerbation of cutaneous symptoms [31]. These reactions may result not only from immediate IgE-mediated responses to cross-reactive allergens, but also from delayed T-cell–mediated mechanisms, whereby even heat-processed foods can trigger worsening of eczematous lesions in predisposed individuals [1,3,25].

### 5.7. Grass Pollens (Poaceae Family)

Across multiple studies, a high prevalence of grass pollen sensitization has been consistently reported among pediatric patients diagnosed with PFAS, although marked regional differences are evident. Grass pollen sensitization is associated with a distinct pattern of cross-reactive foods, involving Cucurbitaceae fruits, particularly watermelon and melon, as well as tropical fruits such as kiwi, citrus fruits, pineapple, and banana. Vegetables, including tomato, potato, and carrot, as well as peanuts and apples, have also been implicated. These cross-reactive responses are primarily mediated by profilins, which function as panallergens across multiple plant species [2,32].

Studies from London demonstrated that 85% of children with pollen–food syndrome were sensitized to grass pollen, with molecular diagnostics revealing specific IgE to the major timothy grass allergens Phl p 1 and Phl p 5 [11]. In the cohort from Sydney, all children with clinically diagnosed PFAS were sensitized to perennial ryegrass pollen, and most were additionally sensitized to other grass species [33]. In East Asia, grass pollen sensitization has also been frequently observed in children with PFAS. A comparative study of Japanese and Korean children showed that sensitization to orchard grass was present in more than half of patients with PFAS and was more common in children with PFAS than in those without PFAS [30], while population-based data from the Tokyo T-Child Study identified sensitization to Phl p 4 in nearly one-third of adolescents with PFAS [18].

Studies have shown that children with PFAS exhibit higher rates of sensitization to profilins compared with pollen-allergic children without food-related symptoms [18]. Sensitization to grass pollen often occurs earlier in childhood than sensitization to birch pollen, potentially explaining the early onset of PFAS symptoms related to grass-associated foods. Early-life exposure to grass pollen, facilitated by its distribution at the height of young children’s breathing zone, has been identified as a risk factor for the subsequent development of PFAS. Sensitization to grass pollen as early as three years of age has been associated with an increased likelihood of PFAS during school age [12].

### 5.8. Weed Pollens (Asteraceae and Related Families)

Ragweed (*Ambrosia* spp.) sensitization among children demonstrates substantial regional variability, with the highest prevalence reported in East Asian populations. In a population-based cohort from Tokyo [18], 32.2% of adolescents with PFAS were sensitized to the ragweed component Amb a 1, compared with 26.6% of pollen-allergic children without PFAS, whereas in Tochigi, where the study population consisted of children with allergic diseases, ragweed sensitization was markedly higher, affecting 81.3% of patients with PFAS and 49.9% of pollen-allergic children without PFAS. Statistical analysis demonstrated that higher levels of ragweed-specific IgE represent a significant risk factor for PFAS. Pollen polysensitization markedly increases PFAS risk, with children sensitized to four pollen types (Japanese cedar, alder, grasses, and ragweed) exhibiting a more than 36-fold higher risk compared with those sensitized exclusively to Japanese cedar [18].

In South Korea, ragweed sensitization has also been reported, particularly in clinical populations of children with atopic diseases, where co-sensitization to ragweed was identified in nearly half of affected patients [31]. Another study from Korea demonstrated that PFAS prevalence increases with the degree of mugwort sensitization, and that children with PFAS who are sensitized to mugwort more frequently present with other atopic comorbidities, including AR and AD [12].

Outside Asia, ragweed sensitization has been well characterized in Italy, where over 20% of children with pollen-induced allergic rhinitis were sensitized, with the highest prevalence observed in the northern regions of the country [34]. In Mexico, ragweed sensitization was observed in 11.6% of children with allergic diseases and was slightly more frequent in those with PFAS [35].

Weed pollen sensitization is associated with characteristic patterns of cross-reactivity with plant-derived foods. Ragweed is most often linked to reactions to melon, watermelon, and banana, whereas mugwort is associated with the mugwort–celery–spice syndrome and reactions to fruits such as peach, melon, and kiwi. Molecular studies suggest that these cross-reactions are frequently mediated by profilins and LTPs, which may contribute to more severe or systemic allergic manifestations in some patients [2,36,37].

Sensitization to mugwort pollen is less frequently responsible for triggering PFAS than tree or other weed pollen. Cross-reactivity is usually mediated by panallergens such as profilins, leading to reactions to foods including celery, peach, melon, and kiwi [28,29].

Data from France suggest a limited role of mugwort in pediatric PFAS, with sensitization reported in only 2.5% of asthmatic children in a Paris cohort [27]. In Mexico mugwort sensitization is uncommon overall; however, isolated associations with PFAS, particularly to peach, have been reported in selected pediatric cases [20]. In central Japan, mugwort sensitization was identified in only 1.6% of children with SAR, also indicating a limited role of this allergen in the general pediatric population [13]. In Tokyo, the prevalence of mugwort sensitization among children with PFAS ranged from 1.6% to 3.4% [18]. However, in an outbreak of OAS following loquat consumption in a school setting in Yamanashi, 41.7% of affected children showed sensitization to ragweed or mugwort, suggesting that mugwort may contribute to PFAS in selected clinical contexts [22].

Available evidence suggests that higher levels of pollen-specific or component-specific IgE are generally associated with an increased likelihood of PFAS manifestations; however, this relationship varies according to the pollen source and the molecular stability of the cross-reactive allergen involved. In birch pollen allergy, higher IgE levels to Bet v 1 are more frequently observed in patients with PFAS than in pollen-sensitized individuals without food-related symptoms [38]. In the nationwide study by Kim et al., the prevalence of PFAS increased progressively with the strength of sensitization to selected pollens, with significant associations observed for birch, oak, and mugwort, suggesting that higher pollen-specific IgE levels may help identify pollinosis patients at increased risk of PFAS [15]. Conversely, sensitization to more stable allergen families, such as lipid transfer proteins or gibberellin-regulated proteins, may be linked to more severe systemic reactions. Therefore, the severity of PFAS appears to depend not only on the magnitude of IgE sensitization to whole pollen extracts, but also on the specific allergenic components involved and their resistance to heat and digestion [1,39].

### 5.9. Food Allergens

The spectrum of food allergens implicated in PFAS in children is strongly influenced by the dominant regional pollens and the underlying cross-reactive pan-allergens. Across studies, several groups of foods have consistently emerged as the most frequent triggers.

Rosaceae fruits represent the classical culprits, particularly in regions with birch or alder pollen allergy. Apples and peaches are most frequently reported, with high prevalence observed in both East Asian and European pediatric populations. In nationwide surveys from Korea, peaches (48.5%) and apples (46.7%) were the leading triggers [15]. Similar associations have been reported in Japan, Croatia, and Italy [14,19,34]. Other Rosaceae fruits, including plums and cherries, are also frequently implicated. These reactions are largely attributed to cross-reactivity with Bet v 1 homologues (PR-10 proteins).

Tropical fruits and members of the Cucurbitaceae family constitute another major group of pediatric PFAS triggers. Kiwifruit is the most common cause in Japanese cohorts (43.6% in Saitama, 39.0% in Tokyo) [16,18] and is also prominent in Italian [34] and Korean children. Pineapple follows closely, being one of the top allergens in Japanese and Korean studies and the leading culprit in Mexico (62.5%) [20]. Melons and watermelons, linked primarily to grass and ragweed pollen sensitization, are common in Japan, Korea, the USA, and Australia, with watermelon being the predominant tropical fruit allergen among Australian children [15,33].

Among vegetables and nuts, tomatoes are notable in East Asia, particularly through cross-reactivity with Japanese cedar pollen (Cry j 1). Peanuts and hazelnuts show strong associations with birch pollen allergy. Peanut was the most frequent food implicated in PFAS-related food allergy in Australian children (57.9%) [33], while hazelnut and walnut were common in Italian and Korean cohorts. Other relevant foods include banana (linked to ragweed cross-reactivity) and avocado, particularly in Mexican populations [22]. Some region-specific foods have been documented. In Korea, indigenous foods such as taro, jujube, ginseng, perilla leaf, and bellflower root were associated with PFAS, reflecting local dietary habits and sensitization patterns [15].

Overall, the prevalence and clinical profile of PFAS in children vary markedly between the general population and cohorts of patients with atopic diseases, with substantially higher rates consistently observed in the latter. These differences are strongly shaped by regional patterns of pollen sensitization, which determine the dominant cross-reactive allergen families and, consequently, the spectrum of triggering foods. Geographic variability in PFAS therefore reflects not only differences in local flora and pollen exposure, but also regional dietary habits and food availability, underscoring the need to interpret pediatric PFAS within both immunologic and environmental contexts.

## 6. Discussion

PFAS in children remains insufficiently characterized from an epidemiological perspective, with available data dominated by studies from East Asia and relatively few population-based investigations from Europe and other regions. Substantial methodological heterogeneity, including variable case definitions (OAS vs. PFAS), different recruitment strategies, and frequent reliance on questionnaire-based symptom reporting without confirmatory diagnostics, limits direct comparison between studies and contributes to wide variability in reported prevalence. Nevertheless, evidence from both general population cohorts and clinical samples indicates that PFAS is more common than previously assumed, particularly among children with allergic rhinitis and polysensitization to pollen.

Clinically, PFAS in childhood is most often characterized by oropharyngeal symptoms, but extra-oral and systemic manifestations are not uncommon and, in a minority of cases, severe reactions including anaphylaxis may occur. This challenges the traditional perception of PFAS as a uniformly mild condition. The syndrome is strongly embedded in a broader atopic phenotype, frequently coexisting with allergic rhinitis and commonly with atopic dermatitis and asthma. Triggering foods and sensitizing pollen vary markedly by geographic region, reflecting differences in local flora, dietary habits, and molecular sensitization profiles. In addition, PFAS can impose a measurable psychosocial burden on children and their families, particularly through dietary restrictions and anxiety related to food safety.

Several limitations should be acknowledged when interpreting the findings of this review. Substantial heterogeneity was observed across the included studies with respect to diagnostic criteria, case definitions, and methods of outcome assessment. PFAS/OAS was variably defined on the basis of self-reported symptoms, questionnaire data, physician diagnosis, sensitization testing, component-resolved diagnostics, or, less frequently, oral food challenges. This methodological variability may have affected prevalence estimates and limited direct comparability between studies. The available evidence is geographically uneven, with most studies originating from selected regions, particularly East Asia and Europe, whereas data from Africa, South America, and several parts of the Middle East remain limited. Given that pollen exposure, dietary patterns, and molecular sensitization profiles differ substantially across regions, this geographic imbalance may restrict the generalizability of the findings. Finally, due to marked heterogeneity in study design, populations, diagnostic approaches, and reported outcomes, a quantitative meta-analysis could not be performed. Therefore, the results were synthesized descriptively, which limits the ability to generate pooled prevalence estimates or formally assess sources of between-study variability.

This review advances current understanding of pediatric PFAS by integrating global evidence on its epidemiology, clinical presentation, atopic comorbidities, food triggers, and molecular sensitization profiles. Unlike earlier reports focused mainly on selected regions or adult populations, it highlights the substantial geographic heterogeneity of PFAS in children and demonstrates how local pollen exposure, dietary habits, and sensitization patterns may shape clinical phenotypes. Clinically, these findings may support earlier recognition of PFAS in children with pollen-induced allergic rhinitis, asthma, or atopic dermatitis, improve risk stratification for systemic reactions, and reduce unnecessary dietary restrictions. The review also identifies key priorities for future research, including standardized diagnostic criteria, harmonized reporting of symptoms and trigger foods, and large, methodologically robust pediatric population-based studies incorporating component-resolved diagnostics to better define the true prevalence, risk factors, and clinical spectrum of PFAS in childhood.

## 7. Conclusions

PFAS is an increasingly recognized but still insufficiently characterized condition in children. Available evidence indicates substantial variability in prevalence, clinical presentation, triggering foods, and sensitization profiles, largely reflecting differences in study populations, diagnostic criteria, geographic pollen exposure, and dietary habits. Although PFAS is usually associated with oropharyngeal symptoms, systemic reactions and anaphylaxis may occur in selected patients, particularly those sensitized to stable allergenic components. Clinicians should consider PFAS in children with pollen-induced allergic rhinitis, asthma, or atopic dermatitis, and diagnostic evaluation should be individualized to distinguish pollen-related cross-reactivity from primary food allergy. Future research should focus on standardized diagnostic criteria, harmonized reporting of clinical outcomes, large population-based pediatric studies, and broader application of component-resolved diagnostics to better define the burden, risk factors, and clinical spectrum of PFAS in childhood.

## Figures and Tables

**Table 1:** Comparison of Class I and Class II Food Allergens.

Feature	Class I Allergens (Primary Food Allergens)	Class II Allergens (Pollen-Related Food Allergens)
Route of sensitization	Gastrointestinal tract (oral exposure)	Respiratory (pollen exposure), followed by cross-reactivity to ingested plant food proteins
Protein stability	High—resistant to heat and digestion (pepsin, low pH, proteolysis)	Low—labile, easily degraded by heat and digestion
Effect of thermal processing	Retain allergenicity after cooking	Usually tolerated after cooking
Clinical manifestations	Systemic reactions might be severe (e.g., anaphylaxis, urticaria, vomiting)	Usually mild, localized reactions (Oral Allergy Syndrome—OAS)
Mechanism of sensitization	Direct IgE response to food proteins	IgE cross-reactivity (pollen–food homologous proteins)
Typical allergenic proteins (examples)	Casein (milk), Ovomucoid (egg), Ara h 1/2/3/6 (peanut), ω-5 gliadin (wheat), Parvalbumin (fish), Tropomyosin (shellfish), Gly m 5 (soy), Ara h 2(peanut), Cor a 9(hazelnut)	PR-10 proteins (Mal d 1—apple, Cor a 1—hazelnut, Api g 1 celery, Gly m 4-soy); Profilins (Mal d 4-apple, Cor a 2-hazelnut, Gly m 3-soy, Api g 4-celery)
Typical food sources	Cow’s milk, egg, peanuts, tree nuts, seeds, soy, wheat, fish, shellfish	Raw fruits (apple, peach, kiwi), vegetables (carrot, celery), nuts (hazelnut, almond), legumes (peanut, soy)
Exceptions	—	Api g 1 (celery), Gly m 4 (soy)—may cause systemic reactions
Special group	Lipid Transfer Proteins (LTPs)—can act as primary allergens, sensitizing via gastrointestinal tract, examples—Mal d 3-apple, Ara h 9-peanut, Cor a 8-hazelnut	LTPs—can also act via cross-reactivity with pollen

**Table 2:** Summary of studies evaluating the prevalence of PFAS OAS in pediatric populations. List of abbreviations: AD—atopic dermatitis, AR—allergic rhinitis, OAS—oral allergy syndrome, PFAS—pollen-food allergy syndrome, SAR—seasonal allergic rhinitis.

Region, Country, Title, First Author	Study Design, Type of Population, Sample Size, Patient Characteristics	PFAS/ OAS Prevalence	The Most Common Pollen Allergens	The Most Common Food Allergens	The Most Common Symptoms	The Most Common Atopic Comorbidity	Key Findings
Japan, Tokyo Pollen-food allergy syndrome and component sensitization in adolescents: A Japanese population-based study, Kiguchi	Cross-sectional birth cohort study, general population, 506, 13-year-olds children.	OAS 16,0% (81/506). PFAS 11.7% (59/506); laboratory-confirmed	Japanese cedar, Cypress, Birch, Alder, Ragweed	Kiwi, Pineapple, Peach, Apple, Tomato, Melon	Oral and throat discomfort, itching (83.1%), swelling of the lips and eyelids (15.3%), facial erythema and urticaria (15.3%)	PFAS population: AR (96.6%) Allergic Conjunctivitis (84.7%) Asthma (35.6%)	PFAS prevalence was 11.7%; kiwi and pineapple were the top causative foods
Japan, Saitama Surveillance of pollen-food allergy syndrome in elementary and junior high school children in Saitama, Japan, Koga	Cross-sectional questionnaire-based study, general population, 2346, schoolchildren 8–13 years old	PFAS 6.9% (156/227)	Japanese cedar, Birch, Timothy grass	Kiwi, Pineapple, Melon, Apple, Peach, Watermelon	Oral symptoms (83.9%) —pain or itching Throat symptoms (49.4%)—itching Ear symptoms (16.7%)	Whole study population: Fruit and vegetable allergy (17.1%) No other specific data concerning allergic conditions.	PFAS prevalence was 6.9%; kiwifruit was the most common causative food (43.6%).
Japan, Osaka Association between fruit and vegetable allergies and pollen-food allergy syndrome in Japanese children: a multicenter cross-sectional case series, Masaaki	Cross-sectional questionnaire-based study, children with food allergy, 97, children 0–15 years old	PFAS 76% (74/97)	Japanese cedar, Birch, Timothy grass	Apple, Peach, Kiwi, Cantaloupe, Watermelon, Cherry, Banana, Pineapple	Itching in the mouth Strange feeling in the throat	Population with food allergy: AR (70%) AD (34%) Asthma (25%)	PFAS accounts for 76% of fruit/vegetable allergy in Japanese children, mostly related to PR-10.
Central Japan, 4 Cities, Regional differences in the prevalence of oral allergy syndrome among Japanese children: A questionnaire-based survey, Ota	Cross-sectional questionnaire-based study, general population, 3365, children 7–15 years old	OAS 15.6% (524/3365)	Japanese cedar, Ragweed, Cypress, Rice	Kiwi, Pineapple, Melon, Japanese yam, Watermelon, Mango, Tomato	Itching, burning, and numbness of the mouth (51.7%) Itching and swelling of the lips (37.4%) Itching, discomfort, and numbness of the throat (18.9%)	Whole study population: SAR (38.1%) Perennial AR (28.8%) Asthma (18.5%) AD (16.3%) OAS population: SAR (59.7%)	Regional differences in OAS prevalence (highest in Maebashi: 21.7%); OAS linked to SAR duration.
Japan, Tochigi Pollen Food Allergy Syndrome in Japanese Children and Adolescents: Risk Factors and Pollen Sensitisation? Kato	Single-center retrospective study, patients with allergies, 600, children and adolescents 3–18 years old	PFAS 20.5% (123/600)	Japanese cedar, Orchard grass, Alder, Ragweed	Peach, Apple, Kiwi, Melon, Pineapple, Strawberry, Tomato, Pear, Watermelon, Orange, Cherry	Tingling. Itching, Oedema of the lips, oral cavity, and/or throat	Whole study population: SAR (77%) AD (48%) Asthma (36%) PFAS population: AR (98.4%) AD (61.8%) Asthma (26%)	PFAS prevalence was 20.5% in patients with pollen allergy, rising to 36.3% in middle-high schoolers.
Japan/Korea, Fukuoka, Tochigi, Busan Relationship among airborne pollen, sensitization, and pollen food allergy syndrome in Asian allergic children, Hwang	Multicenter prospective cross-sectional study, patients with allergies, 133, children 5–17 years old	PFAS Fukoka 25% Tochigi 30% Busan 12%	Japanese cedar, Cypress, Juniper, Orchard grass, Ragweed, Japanese hop, Alder	Kiwi, Cashew nuts, Banana, Peach	No information available	Whole study population: SAR (56%) Food allergy (54%) Asthma (44%) AD (43%)	Alder sensitization is a major determinant of PFAS in children, although sensitization to other pollens was more common.
Japan, Omihachiman Oral symptoms suggestive of oral allergy syndrome in Japanese schoolchildren according to causative food families, Fukao	Questionnaire-based survey, general population, 4991 elementary and junior high school students 6–14 years old	OAS (12.4%) 619/4991	Orchard grass, birch	Kiwi, Melon/Watermelon, Pineapple, Rosaceae (Apple/Peach/Pear)	Oral symptoms	Whole study population: Family history of allergy (44.7%) Present illness of food allergy (3.7%) No other specific data concerning allergic conditions.	Prevalence was higher in females and increased with age.
Japan, Yamanashi Questionnaire Survey on Loquat- Induced Oral Allergy Syndrome in School Children in Yamanashi, Japan, Shimamura	Questionnaire-based survey, general population, 2360 children, including 1768 who consumed loquat; schoolchildren 6–15 years old	OAS after loquat 15% (264/1768) History of OAS 17.2% (407/2360)	Japanese cedar/Cypress, Fagales (Birch, Alder, etc.), Grass, Ragweed	Loquat (newly identified regional trigger), Peach, Apple, Cherry, Kiwi, Melon, Watermelon, Pineapple	Itching or swelling of the oral or pharyngeal mucosa or lips (14.9%) Non-oral symptoms (3.8%)	Whole study population: SAR (75.8%) OAS population: SAR (90.9%)	Prevalence of OAS was highest among upper-grade elementary school students (20.1%), compared with lower-grade students (10.0%) and junior high school students (14.2%).
South Korea Pollen-Food Allergy Syndrome in Korean Pollinosis Patients: A Nationwide Survey, Kim	Nationwide cross-sectional survey, 648 Korean pollinosis patients, including children/adoles cents.	OAS 42.7% (128/300)	Alder, Birch, Beech, Hazel, Oak, Mugwort	Peach, Apple, Kiwi	Oropharyngeal symptoms—tingling/itching sense, oedema (100%) Cutaneus symptoms—pruritus, urticaria and angioedema (43.0%), Respiratory symptoms (20.0%), Gastrointestinal symptoms (10.7%)	Whole study population: AR (90.1%) AC (45.7%) Asthma (39.2%) AD (22.7%) PFAS population: AR (95.9%) AC (55.6%) Asthma (43.3%) AD (29.6%)	The likelihood of PFAS increased with the degree of sensitization (sIgE levels) to birch, oak, and mugwort pollens.
South Korea, Seoul (COCOA Birth Cohort) Food allergy in early childhood increases the risk of oral allergy syndrome in schoolchildren: A birth cohort study, Song	Prospective population-based birth cohort; 930 Korean children aged 6–10 years.	OAS 4.7% (44/930)	Birch, Oak, Alder, Japanese Hop, Ragweed	Kiwi, Peach, Tomato, Watermelon	Itching of the lips, oral cavity, and throat Sore throat Swelling of the lips, oral cavity, or throat	Whole study population: AR (48.9%) AC (16.5%) Asthma (5.1%) AD (16.6%) OAS population: AR (75.6%) Asthma (7.3%) AD (31.7%)	Early childhood food allergy increased school-age OAS risk.
South Korea, Seoul Clinical Characteristics of Oral Allergy Syndrome in Children with Atopic Dermatitis and Birch Sensitization: a Single Center Study, Kim	Retrospective single-center study, children with AD and birch sensitization, 186 children 2–18 years old	OAS 43,5% (81/186)	Birch, Ragweed	Apple, Kiwi, Peach, Pineapple	Oropharyngeal symptoms— Itching (51.9%) Rash (30.9%) Burning sensation: (16.1%) Lip swelling (7.4%) Systemic symptoms (cough, rhinitis, nausea) (3.7%)	Whole study population: AR (90.9%) Asthma (17.2%) OAS population: AR (95.1%) Asthma (21%)	Higher birch-sIgE levels were a significant risk factor.
Sweden Natural course and comorbidities of allergic and nonallergic rhinitis in children, Westman	Population-based birth cohort; 2024 Swedish children followed to age 8 years.	OAS 5% (100/2024)	Birch	Apple, Peach, Kiwi. Banana, Raw carrot	Itching (100%)—diagnostic criterion for OAS in the cohort	Whole study population: AR (5.4% at age 4, 14.0% at age 8) AD (21.3% at age 4, 17.4% at age 8 Asthma (7.5% of children at age 4, 7.3% at age 8)	25% of children with allergic rhinitis had OAS.
Italy Pollen-induced allergic rhinitis in 1360 Italian children: Comorbidities and determinants of severity, Dondi	Nationwide observational survey, 1360, children 4–18 years with pollen-induced allergic rhinitis.	PFAS 23.9% (325/1360) Northern Italy: 30.9% Central Italy: 22.8% Southern Italy and Islands: 16.6%	Timothy grass, Olive tree, Birch, Pellitory, Ragweed	Kiwi, Peach, Apple, Peanut, Hazelnut, Walnut	Oral itching with or without angioedema of the lips or tongue (100%)—diagnostic criterion Urticaria/angioedema (19.9%) Gastrointestinal symptoms (6.62%)	Whole study population: AC (77.1%) Asthma (38.4%) AD (18.5%) PFAS population: AC (84.3%) Asthma (43.8%) AD (19.8%)	Each additional year of allergic rhinitis duration significantly increases the risk of developing PFAS, asthma, and progression to moderate or severe disease.
Italy Endotypes of pollen-food syndrome in children with seasonal allergic rhinoconjunctivitis: a molecular classification, Mastrorilli	Nationwide observational survey, children with seasonal allergic rhinitis and conjunctivitis, 1271 children aged 4–18	PFAS 24% (300/1271) Northern Italy (30.4%), Central Italy (22.2%) Southern Italy and the Islands (16.9%)	Timothy grass, Bermuda grass, Birch Olive, Plane tree, Pellitory, Mugwort	Kiwi, Peach, Apple, Peanut, Hazelnut, Walnut	Oral, pharyngeal, and labial pruritus Angioedema of the lips and tongue Paresthesia of the oral mucosa, palate, and throat	Whole study population: Asthma (38%) AD (36.6%%) PFAS population: Asthma (47%) AD (48%)	Molecular profiles defined distinct clinical endotypes.
Paris, France Emergence of pollen food allergy syndrome in asthmatic children in Paris, Loraund	Retrospective cross-sectional study, asthmatic children, 241, 7–15 years old	PFAS Recent cohort (2012–2018) 8.3% (10/120), Old cohort (1993–1999) 0.8% (1/121)	Birch, Oak, Platanus	Hazelnut, Peanut, Carrot, Kiwi, Apple, Celery, Banana, Soybean.	Oropharyngeal symptoms: Itching, Tingling Angioedema—diagnostic criterion	Whole study population: Recent cohort AR (96%) AD (44%) Old cohort AR (52%) AD (45%)	The prevalence of IgE-mediated food allergy increased from 6% in the earlier cohort to 16% in the contemporary cohort, with this increase being primarily attributable to the emergence of tree pollen–associated PFAS.
Ankara, Turkey Frequency and clinical features of pollen-food syndrome in children, Guvenir	Cross-sectional study, 672 children with SAR, 6–18 years old	PFAS 3.3% (22/672)	Grass pollen	Peach, Kiwi, Tomato, Strawberry, Banana	Swelling of the lips (59%) Pharyngeal pruritus (59%) Oral tingling or irritation (36.4%) Swelling of the tongue (22.7%)	Whole study population: Asthma (45.7%) AD (10.9%) PFAS population: Asthma (36.4%) AD (36.4%)	Significant risk factors for developing PFAS in children were female sex, a history of AD, and a family history of allergic diseases.
Guadalajara, Mexico Prevalence of oral allergy syndrome in children with allergic diseases, Bedolla-Barajasa	Cross-sectional study; 267 children aged 6–14 years with allergic diseases	OAS 8.9% (24/267) AR children 8.8% Asthmatic children 9.1%	Oak, Ash, Prosopis sp.	Pineapple, Peach, Avocado, Banana	Tingling or irritation of the oral cavity (41.7%) Oral itching (41.7%) Throat itching (33.3%) Lip swelling (12.5%)	Whole study population: AR (93.3%) Asthma (69.7%) AD (2.6%)	Tropical fruits, which are typical for the region were the most common causative fruits.
Sydney, Australia The prevalence of the oral allergy syndrome and pollen-food syndrome in an atopic pediatric population in south-west Sydney Cassandra EB Brown	Cross-sectional study, children with atophy, 163 atopic children aged 4–17 years	OAS: 14.7% (24/163) PFAS: 4.9%	Perennial rye, Timothy grass, bermuda grass, birch	Watermelon, kiwi, banana, mango	Labial and oropharyngeal pruritus Paresthesia of the mouth and throat. Angioedema of the tongue, palate, or pharynx Itchy ears. A sensation of throat tightness causing hoarseness.	Whole study population: AR (68.8%) AD (51.5%) Food Allergy (49.7%) Asthma (41.1%) PFAS population: AR (100%) AD (50%) Food Allergy (62.5%) Asthma (87.5%)	Tropical fruits such as watermelon predominated.
London, England Pollen food syndrome amongst children with seasonal allergic rhinitis attending allergy clinic, Ludman	Prospectively recruited cross-sectional study, 54 children with SAR 1–15 years old	PFAS 48% (26/54)	Birch, Timothy grass	Hazelnuts, Apple, Kiwi, Peanut	No information	Whole study population: AD (61%) Food Allergy (61%) Asthma (56%) PFAS population: AD (58%) Food Allergy (50%) Asthma (62%)	Microarray diagnostics has moderate concordance in confirming allergens.
Zagreb, Croatia Oral allergy syndrome in children, Ivkovic-Jurekovic	Prospective observational study; 120 children/adolescents with seasonal AR, 3–18 years old	OAS 26.7% (32/120)	Birch, Grass, Ragweed	Apple, Peach, Carrot, Melon	Oropharyngeal symptoms (97%) Numbness of the lips and itching of the lips, throat, or palate Tightness in the throat (12.5%) Nausea/abdominal pain (3.1%)	Whole study population: AC (91.7%) AD (14.2%) Asthma (45%) OAS population: AC (93.8%) AD (34.4%) Asthma (68.8%)	OAS was significantly more frequent in patients with asthma or atopic dermatitis.
USA A survey on the management of pollen-food allergy syndrome in allergy practices, Songhui Ma	Randomized U.S. allergist survey; 122 responders reporting on children and adults in 2003	OAS children 5% adults 8%	Birch, Ragweed	Apple, Banana, Carrot, Cherry, Citrus, Grape	Itching of the lips, tongue, and oral mucosa Tingling or pulsating sensation in the oral cavity Angioedema of the lips	AR Pollen allergy	Median prevalence of OAS estimates were 5% in children and 8% in adults with pollen allergy.
USA Pollen Food Allergy Syndrome-Two Decades Apart: A Follow-up AAAAI Survey, Wong	Randomized electronic survey; 67 U.S. allergists reporting on pediatric and adult patients in 2023	PFAS children 10% adults 20%	Birch, Ragweed Grass	Fruits, Tree nuts, Peanuts, Celery, Carrot	Itching of the lips, tongue, and oral mucosa Tingling or pulsating sensation in the oral cavity Angioedema of the lips	AR Pollen allergy	Perceived PFAS prevalence in children doubled from 2003 to 2023.

## Data Availability

No new data were created or analyzed in this study.

## Abbreviations

The following abbreviations are used in this manuscript:

AC	Allergic Conjunctivitis
AD	Atopic Dermatitis
AR	Allergic Rhinitis
BA	Bronchial Asthma
CCD	Cross-Reactive Carbohydrate Determinant
CRD	Component-Resolved Diagnostics
GRP	Gibberellin-Regulated Protein
IgE	Immunoglobulin E
LTP	Lipid Transfer Protein
OAS	Oral Allergy Syndrome
OFC	Oral Food Challenge
PFAS	Pollen–Food Allergy Syndrome
PR-10	Pathogenesis-Related Protein 10
SAR	Seasonal Allergic Rhinitis
sIgE	Specific Immunoglobulin E
SPT	Skin Prick Test

## References

[1] Kato Y., Morikawa T., Fujieda S. (2025). Comprehensive review of pollen-food allergy syndrome: Pathogenesis, epidemiology, and treatment approaches. Allergol. Int..

[2] Mastrorilli C., Cardinale F., Giannetti A., Caffarelli C. (2019). Pollen-Food Allergy Syndrome: A not so Rare Disease in Childhood. Medicina.

[3] Alessandri C., Ferrara R., Bernardi M.L., Zennaro D., Tuppo L., Giangrieco I., Ricciardi T., Tamburrini M., Ciardiello M.A., Mari A. (2020). Molecular approach to a patient’s tailored diagnosis of the oral allergy syndrome. Clin. Transl. Allergy.

[4] Price A., Ramachandran S., Smith G.P., Stevenson M.L., Pomeranz M.K., Cohen D.E. (2015). Oral allergy syndrome (pollen-food allergy syndrome). Dermatitis.

[5] Kondo Y., Urisu A. (2009). Oral allergy syndrome. Allergol. Int..

[6] Ferreira F., Hawranek T., Gruber P., Wopfner N., Mari A. (2004). Allergic cross-reactivity: From gene to the clinic. Allergy.

[7] Ballmer-Weber B.K., Vieths S., Lüttkopf D., Heuschmann P., Wüthrich B. (2000). Celery allergy confirmed by double-blind, placebo-controlled food challenge: A clinical study in 32 subjects with a history of adverse reactions to celery root. J. Allergy Clin. Immunol..

[8] Mittag D., Vieths S., Vogel L., Becker W.M., Rihs H.P., Helbling A., Wüthrich B., Ballmer-Weber B.K. (2004). Soybean allergy in patients allergic to birch pollen: Clinical investigation and molecular characterization of allergens. J. Allergy Clin. Immunol..

[9] Ma S., Sicherer S.H., Nowak-Wegrzyn A. (2003). A survey on the management of pollen-food allergy syndrome in allergy practices. J. Allergy Clin. Immunol..

[10] Guvenir H., Dibek Misirlioglu E., Buyuktiryaki B., Zabun M.M., Capanoglu M., Toyran M., Civelek E., Kocabas C.N. (2020). Frequency and clinical features of pollen-food syndrome in children. Allergol. Immunopathol..

[11] Ludman S., Jafari-Mamaghani M., Ebling R., Fox A.T., Lack G., Du Toit G. (2016). Pollen food syndrome amongst children with seasonal allergic rhinitis attending allergy clinic. Pediatr. Allergy Immunol..

[12] Song K.B., Park M.J., Choi E.J., Jung S., Yoon J., Cho H.J., Kim B.S., Ahn K., Kim K.W., Shin Y.H. (2022). Food allergy in early childhood increases the risk of oral allergy syndrome in schoolchildren: A birth cohort study. Pediatr. Allergy Immunol..

[13] Ota M., Nishida Y., Yagi H., Sato K., Yamada S., Arakawa H., Takizawa T. (2023). Regional differences in the prevalence of oral allergy syndrome among Japanese children: A questionnaire-based survey. Asian Pac. J. Allergy Immunol..

[14] Fukao N., Matsumoto A., Motoyama Y., Takeuchi J., Kusunoki T. (2025). Oral symptoms suggestive of oral allergy syndrome in Japanese schoolchildren according to causative food families. Asia Pac. Allergy.

[15] Kim M.A., Kim D.K., Yang H.J., Yoo Y., Ahn Y., Park H.S., Lee H.J., Jeong Y.Y., Kim B.S., Bae W.Y. (2018). Pollen-Food Allergy Syndrome in Korean Pollinosis Patients: A Nationwide Survey. Allergy Asthma Immunol. Res..

[16] Koga T., Tokuyama K., Ogawa S., Morita E., Ueda Y., Itazawa T., Kamijo A. (2022). Surveillance of pollen-food allergy syndrome in elementary and junior high school children in Saitama, Japan. Asia Pac. Allergy.

[17] Buyuktiryaki B., Kulhas Celik I., Erdem S.B., Capanoglu M., Civelek E., Guc B.U., Guvenir H., Cakir M., Dibek Misirlioglu E., Akcal O. (2020). Risk Factors Influencing Tolerance and Clinical Features of Food Protein-induced Allergic Proctocolitis. J. Pediatr. Gastroenterol. Nutr..

[18] Kiguchi T., Yamamoto-Hanada K., Saito-Abe M., Sato M., Irahara M., Ogita H., Miyagi Y., Inuzuka Y., Toyokuni K., Nishimura K. (2021). Pollen-food allergy syndrome and component sensitization in adolescents: A Japanese population-based study. PLoS ONE.

[19] Ivković-Jureković I. (2015). Oral allergy syndrome in children. Int. Dent. J..

[20] Bedolla-Barajas M., Kestler-Gramajo A., Alcalá-Padilla G., Morales-Romero J. (2017). Prevalence of oral allergy syndrome in children with allergic diseases. Allergol. Immunopathol..

[21] Westman M., Stjärne P., Asarnoj A., Kull I., van Hage M., Wickman M., Toskala E. (2012). Natural course and comorbidities of allergic and nonallergic rhinitis in children. J. Allergy Clin. Immunol..

[22] Shimamura A., Kojima R., Ishii H., Matsuoka T., Ikeda H., Sakurai D. (2026). Questionnaire Survey on Loquat-Induced Oral Allergy Syndrome in School Children in Yamanashi, Japan. Allergy.

[23] Bartuzi Z., Kaczmarski M., Czerwionka-Szaflarska M., Małaczyńska T., Krogulska A. (2017). The diagnosis and management of food allergies. Position paper of the Food Allergy Section the Polish Society of Allergology. Postepy Dermatol. Alergol..

[24] Wong L.S.Y., Cox A., Cianferoni A., Katelaris C., Ebo D.G., Konstantinou G.N., Brucker H., Protudjer J.L.P., Hsu Blatman K.S., Boechat J.L. (2026). Pollen Food Allergy Syndrome-Two Decades Apart: A Follow-up AAAAI Survey. J. Allergy Clin. Immunol. Pract..

[25] Al-Shaikhly T., Cox A., Nowak-Wegrzyn A., Cianferoni A., Katelaris C., Ebo D.G., Konstantinou G.N., Brucker H., Yang H.J., Protudjer J.L.P. (2024). An International Delphi Consensus on the Management of Pollen-Food Allergy Syndrome: A Work Group Report of the AAAAI Adverse Reactions to Foods Committee. J. Allergy Clin. Immunol. Pract..

[26] Stiefel G., Anagnostou K., Boyle R.J., Brathwaite N., Ewan P., Fox A.T., Huber P., Luyt D., Till S.J., Venter C. (2017). BSACI guideline for the diagnosis and management of peanut and tree nut allergy. Clin. Exp. Allergy.

[27] Loraud C., de Ménonville C.T., Bourgoin-Heck M., Cottel N., Wanin S., Just J. (2021). Emergence of pollen food allergy syndrome in asthmatic children in Paris. Pediatr. Allergy Immunol..

[28] Kato M., Miyamoto M., Takayanagi F., Ando Y., Fujita Y., Nakayama M., Yoshihara S. (2023). Pollen Food Allergy Syndrome in Japanese Children and Adolescents: Risk Factors and Pollen Sensitisation. J. Immunol. Res..

[29] Hwang Y., Motomura C., Fukuda H., Kishikawa R., Watanabe N., Yoshihara S. (2022). Relationship among airborne pollen, sensitization, and pollen food allergy syndrome in Asian allergic children. PeerJ.

[30] Breiteneder H., Kraft D. (2023). The History and Science of the Major Birch Pollen Allergen Bet v 1. Biomolecules.

[31] Kim K.I., Lee B., Min T.K., Lee J., Pyun B.Y., Jeon Y.H. (2019). Clinical Characteristics of Oral Allergy Syndrome in Children with Atopic Dermatitis and Birch Sensitization: A Single Center Study. J. Korean Med. Sci..

[32] Krikeerati T., Rodsaward P., Nawiboonwong J., Pinyopornpanish K., Phusawang S., Sompornrattanaphan M. (2023). Revisiting Fruit Allergy: Prevalence across the Globe, Diagnosis, and Current Management. Foods.

[33] Brown C.E., Katelaris C.H. (2014). The prevalence of the oral allergy syndrome and pollen-food syndrome in an atopic paediatric population in south-west Sydney. J. Paediatr. Child Health.

[34] Dondi A., Tripodi S., Panetta V., Asero R., Businco A.D., Bianchi A., Carlucci A., Ricci G., Bellini F., Maiello N. (2013). Pollen-induced allergic rhinitis in 1360 Italian children: Comorbidities and determinants of severity. Pediatr. Allergy Immunol..

[35] Gilles S., Akdis C., Lauener R., Schmid-Grendelmeier P., Bieber T., Schäppi G., Traidl-Hoffmann C. (2018). The role of environmental factors in allergy: A critical reappraisal. Exp. Dermatol..

[36] Hauser M., Roulias A., Ferreira F., Egger M. (2010). Panallergens and their impact on the allergic patient. Allergy Asthma Clin. Immunol..

[37] Popescu F.D. (2015). Cross-reactivity between aeroallergens and food allergens. World J. Methodol..

[38] Olcese R., Silvestri M., Del Barba P., Brolatti N., Barberi S., Tosca M.A., Ciprandi G. (2019). Mal d 1 and Bet v 1 sensitization pattern in children with Pollen Food Syndrome. Allergol. Int..

[39] Jeong K., Lee S. (2025). Update on Tree Nut and Seed Allergies: Prevalence, Clinical Characteristics, Diagnosis, and Management. Allergy Asthma Immunol. Res..

